# Intensified concurrent chemoradiotherapy with 5-fluorouracil and irinotecan as neoadjuvant treatment in patients with locally advanced rectal cancer

**DOI:** 10.1038/sj.bjc.6602492

**Published:** 2005-03-22

**Authors:** G Klautke, P Feyerherd, K Ludwig, F Prall, T Foitzik, R Fietkau

**Affiliations:** 1 Department of Radiotherapy, University Hospital, Südring 75, 18059 Rostock, Germany; 2 Department of Surgery, University Hospital, Rostock, Germany; 3 Department of Surgery, Klinikum Südstadt, Rostock, Germany; 4 Department of Pathology, University Hospital, Rostock, Germany

**Keywords:** rectal cancer, neoadjuvant chemoradiotherapy

## Abstract

This study aimed to evaluate the feasibility and efficacy of neoadjuvant chemoradiotherapy intensified with irinotecan in patients with locally advanced rectal cancer. Eligible patients had nonmetastatic disease at a locally advanced stage that made R0 resection and sphincter preservation uncertain. They received preoperative radiation over 6 weeks to 45 Gy and boost of 5.4 Gy and concurrent continuous infusion 5-fluorouracil 250 mg m^−2^ day^−1^ and weekly irinotecan 40 mg m^−2^. In all, 37 patients entered the study. T stage at baseline as determined by ultrasound was T2/T3/T4 in 2/19/16 patients; 31 patients had lymph node involvement. The predominant toxicity was diarrhoea (grade 3/4 in 10/2 patients). Haematologic toxicity and surgical complications were moderate. Among 36 patients undergoing surgery, 32 (89%) had R0 resection and 23 (64%) sphincter preservation. Pathologic complete response (pCR) was achieved in eight (22%) of 36 patients, and 10 patients (28%) had only microscopic residual disease. At 4 years, overall survival was 66%, disease-free survival 73%, local relapse rate 7%, and distant failure rate 24%. Extent of resection and postoperative nodal status were significant predictors of overall and disease-free survival. Intensified neoadjuvant chemoradiotherapy with irinotecan can be safely administered and results in a high pCR rate.

It is of critical prognostic significance in rectal cancer whether or not a resection of the tumour with clear margins (R0) can be achieved (Hermanek and Wittekind, 1994). On the other hand, maintenance of foecal continence is of crucial importance for the patients' quality of life. Even if state-of-the art surgical techniques are used, often both of these aims cannot be accomplished in locally advanced tumours, particularly if they are located in the lower third of the rectum. Several phase II studies of 5-fluorouracil-based preoperative chemoradiotherapy for locally advanced rectal cancer have reported R0 resection rates of 60–85% (Chan *et al*, 1993; Minsky *et al*, 1993; Keilholz *et al*, 1995; Videtic *et al*, 1998; Küchenmeister *et al*, 2000; Rödel *et al*, 2000) including pathologic complete response (pCR) rates of 5–20%. Even in low rectal tumours, sphincter-preserving surgery was possible after preoperative chemoradiotherapy in 27–86% of the patients (Grann *et al*, 1997; Hyams *et al*, 1997; Maghfoor *et al*, 1997; Küchenmeister *et al*, 2000). Moreover, randomised trials have shown that neoadjuvant chemoradiotherapy improves local control compared with postoperative chemoradiation in primary resectable tumours (Roh *et al*, 2001; Sauer *et al*, 2004). The probability of distant metastasis, however, is not reduced. In addition to the well-established agent 5-fluorouracil, new agents such as irinotecan or oxaliplatin have proven effective in the treatment of metastatic colorectal cancer. Therefore, the present phase II study was aimed to determine the feasibility and local efficacy of an intensified neoadjuvant treatment approach using concurrent radiation and chemotherapy with 5-fluorouracil and irinotecan in patients with locally far advanced rectal cancer.

## PATIENTS AND METHODS

### Patient eligibility

Male and female patients with histologically confirmed adenocarcinoma of the rectum were prospectively enrolled in the study if they presented with nonmetastatic disease at a locally advanced stage that made R0 resection and sphincter preservation uncertain. Other eligibility criteria included measurable disease (at least one bidimensionally measurable tumour lesion), WHO performance status ⩽2, adequate haematologic, hepatic and renal function, and life expectancy of at least 3 months. Pregnant or lactating women, patients with unresolved bowel obstruction or ileus/subileus, and those with a history of chronic diarrhea were ineligible for study entry. All patients underwent baseline examination and staging within 2–4 weeks prior to the start of chemoradiotherapy, including history and physical examination, complete blood count, serum chemistry profile, chest X-ray, rectoscopy or sigmoidoscopy, endoluminal ultrasound, abdominal ultrasound, and computed tomography (CT) of the abdomen and pelvis. Inclusion of approximately 35 patients was planned. The study was approved by the local ethics committee, and written informed consent was obtained from all patients.

### Treatment

Computed tomography-assisted three-dimensional planning of radiation therapy was employed. The patients underwent CT with 5 mm slices, contrast administration to bladder, rectum and small intestine, and endoscopic clipping (Riepl *et al*, 2000) of the upper and lower borders of the tumour performed immediately prior to planning. Radiation therapy was given with photons from a linear accelerator with energy >6 MV. The target volume comprised the areas at risk including the presacral space along the posterior bladder or vaginal wall, respectively, and the common iliac lymph nodes until and including the fifth lumbar vertebral body. Radiotherapy was delivered with three or four fields using an isocentric technique with individually collimated field portals. Daily fractions of 1.8 Gy (isodose 90% of the maximum dose) were given on 5 days a week over 5 weeks to a total dose of 45 Gy (isodose 90%, corresponding to 47.5–48 Gy as calculated according to the International Commission on Radiation Units and Measurements (ICRU) reference point approach). An additional low-volume boost of 5.4 Gy (5.8 Gy by ICRU reference point approach) was given in three fractions on days 1–3 of week 6 of treatment to the site of the primary tumour after previous contrast radiography of the small intestine.

Radiation was administered with concurrent chemotherapy that consisted of 5-fluorouracil given by continuous infusion via an implantable port system at a daily dose of 250 mg m^−2^ throughout the entire treatment period (days 1–43). In addition, irinotecan was administered once weekly at 40 mg m^−2^ by 90-min infusion immediately prior to the first weekly fraction of radiation. Premedication with atropin 0.25 mg subcutaneously was recommended to prevent irinotecan-associated acute cholinergic syndrome. Irinotecan doses were omitted if leucocyte or platelet nadir values were less than 2000 *μ*l^−1^ or <75 000 *μ*l^−1^, respectively, or if grade 4 toxicity occurred.

Restaging and surgery using total mesorectal excision was to be performed within 4–6 weeks after completion of chemoradiotherapy. Following surgery, patients usually received adjuvant chemotherapy with 5-FU with or without folinic acid according to the recommendations of the German Cancer Society (DKG).

### Data evaluation

The deadline for data evaluation was December 31, 2003. Statistical analysis including survival analysis according to Kaplan–Meier was performed with the SPSS software package. Survival was calculated from the date of histologic verification of diagnosis to the patient's death or the date of last follow-up. Progression-free survival was calculated from diagnosis to the time of first detection of new lesions or progression of residual lesions. Toxicity was graded according to the National Cancer Institute Common Toxicity Criteria (CTC) modified by Seegenschmiedt (1998).

## RESULTS

### Patient characteristics

From July 1, 1999 to December 31, 2001, a total of 37 patients (10 females, 27 males) aged from 41–77 years (median, 62 years) were referred from the surgeons with locally advanced rectal cancer without distant metastasis to our department. All these patients, fulfilling the including criteria, without any selection were enrolled in the study. The main reason for the referral of this patients was an inquestionable R0 resection without preoperative treatment, because of great tumour mass in the pelvis – the median longitudinal extension of the tumour based on endorectal ultrasound was 6 cm (range 3–13) – or the infiltration of other organs. In two cases the main reason was a potentially shincter preservation by tumours located in the lower third of the rectum (0–3 cm, and 1–3 cm from the anal verge). The patient characteristics are shown in Table 1. Most tumours were located in the lower third of the rectum (22 patients); in 13 patients the tumour affected the middle third (5.5–10 cm from the anal verge) and in two patients the upper third of the rectum (>10 cm from the anal verge).

### Feasibility and toxicity

Six doses of irinotecan were given once weekly as planned in 25 (68%) of all 37 patients. One and two doses of irinotecan had to be omitted in nine (24%) and three (8%) patients, respectively, in three cases because of leukopenia grade 3 and 4, in three cases because of fever, and in six cases because of diarrhoea grade 3 and 4. Acute cholinergic symptoms including acute diarrhoea were not encountered, and premedication with atropin was not required. Thromboembolic events were also not seen. Haematologic toxicity was moderate. Thrombocytopenia grade 3 occurred in one patient (2%), and leukopenia grade 3 and 4 in three (8%) and one (2%) patient, respectively. Anaemia grade 3/4 was not observed. Haemoglobin fell from a median baseline value of 13.2 g dl^−1^ (range 10.1–16.7) to a median nadir of 11.6 g dl^−1^ (range 9.2–15.2) during treatment and reincreased to 12.2 g dl^−1^ (range 9.7–15.8) at completion of chemoradiotherapy. The predominant nonhaematologic toxicity was delayed diarrhoea that reached grade 3 in 10 patients (27%) and grade 4 in two patients (5%). Patients with grade 4 diarrhoea were hospitalised and received parenteral nutrition. One patient developed an extrapontine myelinolysis due to severe electrolyte imbalance 2 weeks after completion of therapy; she received appropriate treatment and recovered without sequelae. Three patients (8%) developed fever that could be easily controlled with usual antibiotics. Oral mucositis was not observed, and also no nausea and vomiting grade 2 and higher, because all patients received before each application of irinotecan dexamethasone 8 mg and ondansetron 8 mg in a prophylactic way. Abdominal pain, as one criteria in the definition of the grade of diarrhoea in the CTC, was observed in seven patients. Overall, intensified chemoradiotherapy was very well tolerated. Minor complications included transient bladder dysfunction and urinary retention in two patients that resolved completely without any therapy, however, within 2–3 weeks after surgery as a typical side effect of surgery. There were two anastomotic strictures without any symptoms stumbled on 3 months after primary surgery, which required dilatation prior to ileostomy closure. In addition, anastomotic leakage that was amenable to conservative treatment occurred in two patients, and one perianal wound infection with subsequent secondary healing.

In one patient chemoradiotherapy had to be discontinued after 40 Gy for signs and symptoms of an acute abdomen. A stenosis of the small intestine was detected in the radiation field in this patient; he underwent surgery during the acute phase, and subsequent reoperation was necessary due to an anastomotic leak.

### Resectability and type of surgery

Of the 37 patients, 36 proceeded to surgery after completion of neoadjuvant chemoradiotherapy. One patient refused surgery because an exploratory biopsy had shown no evidence of residual tumour. An R0 resection was possible in 32 (89%) of the 36 patients undergoing surgery; the four (11%) remaining patients had an R1 resection. Sphincter preservation could be achieved in 23 (64%) of 36 patients, while a radical resection of the rectum was required in 13 (36%) patients. Table 2 shows the surgical approach by tumour height. So sphincter-saving surgery was possible in 10 of 22 patients (45%) with a tumour less or 5 cm from the anal verge, and in 21 of 34 patients (61%) with a tumour less or 10 cm from the anal verge.

### Pathologic response and downstaging

Histologic examination of the resection specimens demonstrated a pCR of the primary tumour in eight of 36 patients (22%). The inclusion of the patient who had only an exploratory biopsy increased the pCR rate to 24% (nine out of 37). In all, 10 patients (28%) had only microscopic residual disease (MRD), and 14 patients (39%) had a tumour regression by more than 50%, resulting in a pathologic partial response rate of 67%. A minor response was achieved in four (11%) patients. No patient failed to respond. Tumour downstaging by at least one T category was achieved in 28 (76%) of all 37 patients (Table 3). The pathologic lymph node status at the time of surgery was pN0 in 25 of 36 patients (69%), pN1 in five patients (14%), and pN2 in six patients (17%).

### Disease control and survival

At a median follow-up of 40 months (range 28–53), 10 of 37 patients had died, of which eight succumbed to their disease. One patient died after 5 months of pulmonary embolism and another one after 2 years of congestive heart failure. The estimated local relapse rate (±standard deviation) at 4 years was 7±5%. One patient developed an isolated local recurrence, and another patient had synchronous local recurrence and liver metastasis. The rate of distant metastasis at 4 years was 24±7%. Among patients with R0 resection, the rate of distant metastasis was 14±6.3%. The actuarial progression-free and overall survivals at 4 years were 73±7.6 and 66±8.6%, respectively, the disease-specific overall survival at 4 years was 70±8.7%.

**Figure 1:**
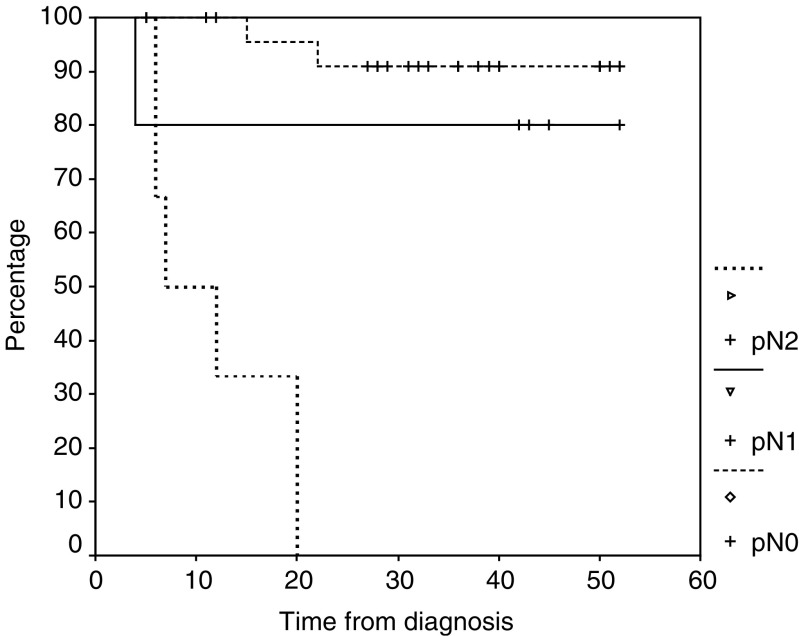
Progression-free survival by nodal status: pN0, 25 patients (4-year PFS 92±6.1%); pN1, five patients (4-year PFS 80±17.9%); pN2, six patients (4-year PFS 0%). *P*<0.0001.

**Figure 2:**
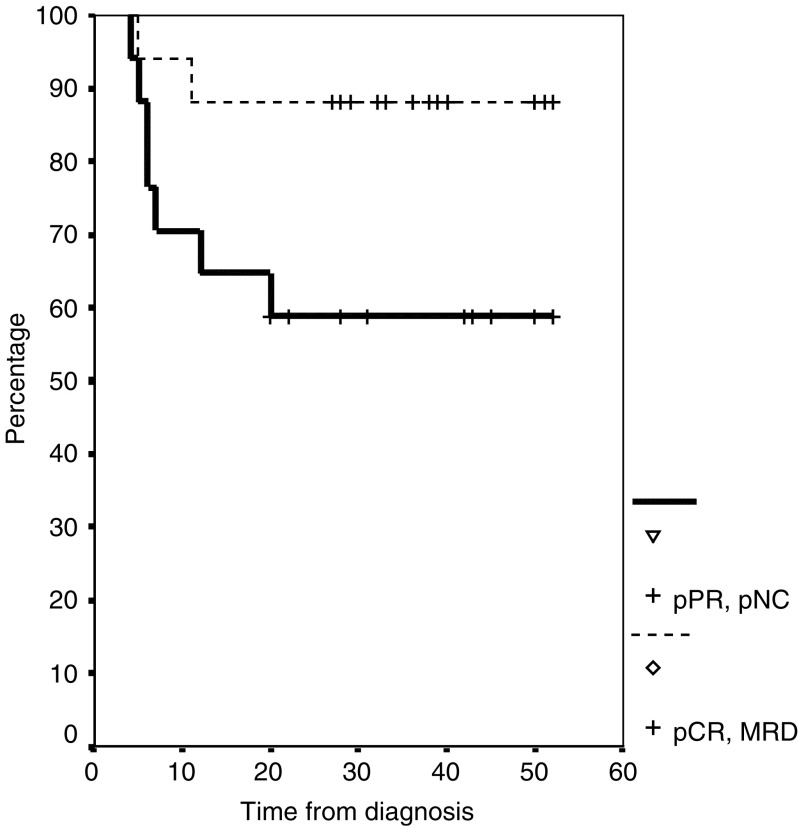
Progression-free survival by response status: pCR, MRD: 18 patients (4-year PFS 88±8%); pPR, pNC 18 patients (4-year PFS 58±12%); *P=*0.06.

Among postoperative disease variables, the resection (R) and nodal (N) status and the pathohistological response rate were found to be the most important prognostic determinants. Overall survival at 4 years was 81±7.1% among patients with an R0 resection compared with 0% among those who had an R1 resection (*P=*0.013). Disease-specific survival rates at 4 years were 84±6 and 0% for the R0 and R1 patient subgroups, respectively (*P=*0.0001). Patients with no pathologic evidence of lymph node involvement (pN0) after neoadjuvant chemoradiotherapy had a 4-year progression-free survival of 92±6.1% (Figure 1) compared with 80±17.9% for patients with pN1 and 0% for those with pN2 (*P*<0.0001). Overall survival at 4 years was 80% among patients with pN0 or pN1 status compared with 33% among patients with pN2 (*P=*0.01). The progression-free survival at 4 years for patients with complete responses or only MRD was 88±8% (Figure 2) and for patients with only partial response 58±12% (*P=*0.06).

## DISCUSSION

The primary aim of treatment for rectal cancer is to achieve complete resection of the tumour and, in the long term, a high local control and low distant failure rate. An additional aim in low rectal tumours is sphincter preservation. These objectives are difficult to meet in advanced stages of the disease, and treatment outcome often remains unsatisfactory in this setting. To improve the prognosis compared with surgery alone, neoadjuvant 5-fluorouracil-based chemoradiotherapy has been introduced, with 5-fluorouracil given as a bolus or continuous infusion, with or without folinic acid. Although these studies reported high rates of R0 resection, pCR, and sphincter saving, even in low tumours, many patients eventually developed local or, more common, distant recurrences. Intensification of neoadjuvant therapy appears a promising approach to eradicate potential micrometastases more effectively prior to surgery. Since new agents have improved treatment results in the palliative setting, several phase II studies have recently been initiated to examine the usefulness of these agents given concurrently with radiotherapy as neoadjuvant treatment. The most promising of these agents are oxaliplatin and irinotecan. We added irinotecan to 5-fluorouracil in our study for two reasons: Owing to its high activity in advanced colorectal cancer (Douillard *et al*, 2000; Saltz *et al*, 2000) irinotecan appears particularly suited for intensified induction therapy; in addition, several studies have documented the radiosensitising properties of irinotecan (Boothmann *et al*, 1987; Boscia *et al*, 1993; Omura *et al*, 1997; Chen *et al*, 1999) that were observed even under hypoxic conditions (Boscia *et al*, 1993). As hypoxia may have an adverse effect on the radiosensitivity of cells (Kumar, 2000; Fyles *et al*, 2000), irinotecan may be particularly useful as a constituent of chemoradiotherapy programmes in patients with bulky pelvic tumours and poor blood supply that often contain hypoxic regions.

The R0 resection rate of 89% in our study is in the upper range of results reported for neoadjuvant chemoradiotherapy in locally advanced rectal cancer with 5-fluorouracil alone. Moreover, our long-term local control rate of 93% at 4 years is comparable to the results achieved with concurrent 5-fluorouracil and radiation in the study of the German Rectal Cancer Group (Sauer *et al*, 2004), even though patients in this study had on average less advanced disease. Sphincter-saving surgery was possible in our study in as many as 10 (45%) out of 22 patients with very low rectal cancers (⩽5 cm from anal verge), most of which would have required total resection if no neoadjuvant treatment had been given. One of these 10 patients developed after 15 months distant metastasis in the liver and a locoregional recurrence, and died after 39 months with normal sphincter function.

The intensified neoadjuvant multi-modality programme with 5-fluorouracil, irinotecan, and concurrent radiation that we used in our study shows a higher rate of diarrhoea compared with studies using only 5-fluorouracil in different ways. So, in schemes with only 5-fluorouracil, the rate of grade ¾ diarrhoea is about 20–25%; adding irinotecan, the rate of grade ¾ diarrhea is about 30–40%. In our study the rate of grade ¾ diarrhoea was 32% and this is in keeping with the findings in other studies using this combination (Mitchell *et al*, 2001; Mehta *et al*, 2003). Therefore, this intensified neoadjuvant scheme with 5-fluorouracil, irinotecan, and concurrent radiation must include an intensified care and supportive treatment for the patients. Then this scheme is safe and effective. Since both 5-fluorouracil and pelvic radiation cause diarrhoea as an adverse effect, irinotecan cannot be administered at weekly doses of 80–100 mg m^−2^ that are usually given in combination with 5-fluorouracil and folinic acid without radiation therapy (Douillard *et al*, 2000; Saltz *et al*, 2000). We chose an irinotecan dose of 40 mg m^−2^, while 50 mg m^−2^ was used in the other published studies, though in combination with a lower daily dose of 5-fluorouracil (225 mg m^−2^) compared with our regimen (250 mg m^−2^). The total doses of irinotecan were 200 and 250 mg m^−2^ in the other studies (Mitchell *et al*, 2001; Mehta *et al*, 2003) and 240 mg m^−2^ in the present trial. Diarrhoea was also a predominant nonhaematologic toxicity seen with other chemoradiation protocols (Table 4). Nevertheless, adding irinotecan to radiation and 5-fluorouracil the rate of diarrhoea is higher, but this acute toxicity is manageable with an intensified care and if needed with supportive treatment.

The effectivity of neoadjuvant therapy of rectal cancer is usually measured by the rate of pathologically confirmed complete responses (pCR). A pCR rate of 8–10% was found in studies with 5-fluorouracil-based regimens, 5% in an earlier study of our group (Küchenmeister *et al*, 2000) and the substitution of an oral prodrug for intravenous 5-fluorouracil does not appear to improve these results, with a pCR rate of 4–9% (Table 4) besides one exception by a group from Korea with a pCR rate of 31% (Kim *et al*, 2002). Compared with these data, the addition of irinotecan to 5-fluorouracil in the present study resulted in a gross doubling of the pCR rate to over 20%. These favourable results are in line with those recently reported for the 5-fluorouracil–irinotecan combination by Mehta *et al* (2003) and Mitchell *et al* (2001), who achieved a pCR rate of 37% in 32 patients with T3 tumours, and of 24% in a patient population which included a few T4 tumours, respectively (Table 4). More recently, pCR rates from 12 to 21% were reported for the combination of oxaliplatin with 5-fluorouracil or 5-fluorouracil prodrugs.

In the preoperative treatment of other gastrointestinal cancers, like gastric cancer or oesophageal cancer, the rate of pCR is an independent factor for survival (Forastiere *et al*, 1997; De Vita *et al*, 2002; Terrosu *et al*, 2003; Ajani *et al*, 2004). Also, in our study, the progression-free survival for patients with complete responses or only MRD is higher than for patients with only partial response (4-year OS: 88±8 *vs* 58±12%; *P=*0.06; see Figure 2).

Unfortunately, most studies of intensified neoadjuvant chemoradiotherapy did not report long-term results with regard to local control, late toxicities or, most importantly, the probability of distant metastasis. We have therefore evaluated our data after a long median follow-up of 36 months, with a minimum follow-up of 24 months for surviving patients. In randomised trials, well over 30% of the patients with stage II or III rectal cancer developed distant dissemination. However, neoadjuvant chemoradiation regimens including 5-fluorouracil alone have failed to prevent tumour spread to distant organs. The size of the problem related to distant metastasis in locally advanced rectal cancer becomes even more evident if one considers not only selected study populations but also consecutive patient series in everyday practice. In a retrospective analysis of patients who received adjuvant chemoradiotherapy with 5-fluorouracil and folinic acid at the University of Würzburg, Germany, distant failures occurred in as much as 49% of stage III patients (Bagatzounis *et al*, 2000). Although the patients in our phase II study had comparatively large, advanced tumours, only 24% developed distant metastasis, and among those with R0 resection the rate was as low as 14%. Taking also into account the extended follow-up, these results possibly suggest an improved systemic effect of our intensified neoadjuvant treatment regimen compared with the other adjuvant or neoadjuvant protocols reported so far. Of course these data has to be proven in phase III trials comparing neoadjuvant 5-fluorouracil-based treatment with intensified schemes with oxaliplatin or irinotecan.

Another interesting, though preliminary, finding of our study was the outstanding prognostic significance of postoperative nodal status. While pN0 and pN1 patients had progression-free survivals at 4 years of 92 and 80%, respectively, all pN2 patients had a recurrence, usually at a distant site, within the first 2 years (Figure 1). It is likely therefore that the former group of patients will not benefit from further intensification of treatment. In contrast, additional postoperative therapy, perhaps in the form of maintenance chemotherapy, should be strongly considered in pN2 patients.

## Figures and Tables

**Table 1:** Baseline patient characteristics

**Variable**	**Patients (*N*=37)**	
	**No.**	**%**
*Gender*		
Male	27	73
Female	10	27
		
*Age (years)*		
Median	62	
Range	41–77	
		
*T stage*		
uT2	2	5
uT3	19	51
uT4	16	43
		
*N stage*		
uN0	6	16
uN+	31	84
		
*Tumour localisation (cm from anal verge)*		
0–5	22	59
5.5–10	13	35
>10	2	5

**Table 2:** Surgical approach by tumour height

**Tumour height (cm)**	**Surgical approach (No. of patients)**		
	**Sphincter-saving**	**Radical**	**Total**
0–5	10	12	22
5.5–10	11	1	12
>10	2	0	2

**Table 3:** Pathologic downstaging of the primary tumour

**Clinical T stage at baseline**	**Pathologic T stage at the time of surgery (patients)**					
	**pT0**	**pT1**	**pT2**	**pT3**	**pT4**	**Total**
cT2	1	0	0	1	0	2
cT3	4	1	7	7	0	19
cT4	4	0	2	9	1	16
Total	9	1	9	17	1	37

**Table 4:** Overview of preoperative chemoradiotherapy with different chemotherapeutics

**Study**	**No. of patients**	**Chemotherapy**	**RT dose**	**Diarrhoea III/IV**	**PCR (%)**
*5-Fluorouracil*					
Sauer *et al*, 2004	421	5-FU	50.4	12%	8
Roh *et al*, 2001	130	5-FU/LV		Grade 4 24%	10
					
*Oral 5-FU prodrugs*					
Fernandez-Martos *et al*, 2004	94	UFT	45	14%	9
Dunst *et al*, 2004	98	Capecitabin	50.4+5.4	4%	4
Kim *et al*, 2002	45	Capecitabin/LV	45+5.4	4%	31
					
*5-FU/oxaliplatin*					
Gerard *et al*, 2003	40	5-FU/oxaliplatin	50	17%	15
Pinto *et al*, 2004	26	5-FU/oxaliplatin	45+5.4	14%	12
					
*Oral 5-FU prodrugs/oxaliplatin*					
Rodel *et al*, 2003	32	Capecitabin/oxaliplatin	50.4	12%	19
Glynne-Jones *et al*, 2004	86	Capecitabin/oxaliplatin	45	10%	21
					
*5-FU/Irinotecan*					
Mitchell *et al*, 2001	49	5-FU/Irinotecan		30%	24
Metha *et al*, 2003	32	5-FU/Irinotecan	50.4	28%	37
					
*Oral 5-FU prodrugs/irinotecan*					
Hofheinz *et al*, 2004	19	Capecitabin/irinotecan	45+5.4	16%	21
Klautke *et al*, 2004	23	Capecitabin/irinotecan	50.4+5.4	39%	18
